# A Polygenic Risk Score to Predict Future Adult Short Stature Among Children

**DOI:** 10.1210/clinem/dgab215

**Published:** 2021-03-31

**Authors:** Tianyuan Lu, Vincenzo Forgetta, Haoyu Wu, John R B Perry, Ken K Ong, Celia M T Greenwood, Nicholas J Timpson, Despoina Manousaki, J Brent Richards

**Affiliations:** 1 Lady Davis Institute for Medical Research, Jewish General Hospital, Montréal, Canada; 2 Quantitative Life Sciences Program, McGill University, Montréal, Canada; 3 Department of Epidemiology, Biostatistics and Occupational Health, McGill University, Montréal, Canada; 4 Medical Research Council Epidemiology Unit, Institute of Metabolic Science, University of Cambridge, Cambridge, UK; 5 Department of Pediatrics, University of Cambridge School of Clinical Medicine, Cambridge, UK; 6 Department of Human Genetics, McGill University, Montréal, Canada; 7 Gerald Bronfman Department of Oncology, McGill University, Montréal, Canada; 8 Medical Research Council Integrative Epidemiology Unit, Department of Population Health Sciences, University of Bristol, Bristol, United Kingdom; 9 Department of Pediatrics, Université de Montréal, Montréal, Canada; 10 Department of Twin Research and Genetic Epidemiology, King’s College London, London, UK

**Keywords:** polygenic risk score, adult height prediction, short stature, parental height, UK Biobank, ALSPAC

## Abstract

**Context:**

Adult height is highly heritable, yet no genetic predictor has demonstrated clinical utility compared to mid-parental height.

**Objective:**

To develop a polygenic risk score for adult height and evaluate its clinical utility.

**Design:**

A polygenic risk score was constructed based on meta-analysis of genomewide association studies and evaluated on the Avon Longitudinal Study of Parents and Children (ALSPAC) cohort.

**Subjects:**

Participants included 442 599 genotyped White British individuals in the UK Biobank and 941 genotyped child-parent trios of European ancestry in the ALSPAC cohort.

**Interventions:**

None.

**Main Outcome Measures:**

Standing height was measured using stadiometer; Standing height 2 SDs below the sex-specific population average was considered as short stature.

**Results:**

Combined with sex, a polygenic risk score captured 71.1% of the total variance in adult height in the UK Biobank. In the ALSPAC cohort, the polygenic risk score was able to identify children who developed adulthood short stature with an area under the receiver operating characteristic curve (AUROC) of 0.84, which is close to that of mid-parental height. Combining this polygenic risk score with mid-parental height or only one of the child’s parent’s height could improve the AUROC to at most 0.90. The polygenic risk score could also substitute mid-parental height in age-specific Khamis-Roche height predictors and achieve an equally strong discriminative power in identifying children with a short stature in adulthood.

**Conclusions:**

A polygenic risk score could be considered as an alternative or adjunct to mid-parental height to improve screening for children at risk of developing short stature in adulthood in European ancestry populations.

Predicting adult height is important to monitoring childhood growth and development, particularly for children in some jurisdictions who may be considered for growth hormone therapy for “idiopathic short stature” (1-3). Various approaches have been developed and clinically adopted for adult height prediction, based on current height measures and bone age as measured by hand and wrist X-ray, such as the Bayley-Pinneau method (4), the Tanner-Whitehouse methods (5,6), and the Roche-Wainer-Thissen method (7). While bone age has been recognized as one of the most important predictors of final adult height (8) and has been employed in these prediction approaches, measuring bone age requires an X-ray and a radiologist’s interpretation of the images and calculation of the predicted adult height. Also, in some medical conditions affecting bone structure (such as glycogen storage diseases (9)), determination of bone age from X-ray can be challenging.

Since height is highly heritable, with an estimated heritability of approximately 80% (10,11), efforts have been made to genetically predict height. In the 19th century, the concept of mid-parental height was proposed (12). It has been either used directly as an empirical predictor of adult height or incorporated into other height prediction methods, such as the bone age-free Khamis-Roche method (13), to represent the genetic contribution to an individual’s adult height. Nevertheless, mid-parental height has a few noteworthy limitations. First, it is a surrogate of genetic effects and does not reflect the exact number of height-influencing alleles inherited from the parents. Consequently, its accuracy as a predictor decreases when the precision of the measurement of parental heights decreases. This can occur when the parents’ height is estimated or measured after it has started declining from peak adult height. Further, when one or both biological parents’ heights are unknown (which often happens for adopted children and nonnuclear families or when a parent is deceased) calculating mid-parental height may be impossible. Hence, finding alternative and more accurate genetic predictors of adult height could be helpful to clinicians.

Directly assessing the presence of alleles that influence height has become practical and relatively low cost through the use of genomewide genotyping. With large genotyped cohorts, such as the UK Biobank (14), polygenic risk scores for complex traits of high heritability and polygenicity have been developed and achieved high predictive and discriminative power (15-18). Although it has been shown that at least 3290 near-independent genomewide significant single nucleotide polymorphisms (SNPs) are associated with adult height (19), a recently constructed polygenic risk score has been able to explain about 40% of the total variance in sex-adjusted height z-scores (20).

In this study, we sought to develop and optimize a polygenic risk score for adult height using state-of-the-art methods based on 607 346 individuals of European ancestry from the UK Biobank and the Genetic Investigation of Anthropometric Traits (GIANT) study (21). We then evaluated how well such a polygenic risk score performed compared to mid-parental height in its ability to predict adult height of 941 children from the Avon Longitudinal Study of Parents and Children (ALSPAC) (22,23). Last, we tested the ability of the polygenic risk score to identify children who would later have an adulthood short stature.

## Methods

### Study Cohorts

The UK Biobank study recruited more than 500 000 participants who were enrolled between 2006 and 2010 and were aged between 40 and 69 years, at multiple recruitment centers in the United Kingdom (14). Demographic and anthropometric measurements were collected upon recruitment (Table 1). Shoeless standing height was measured using a Seca 202 mechanical telescopic height measuring rod. Participants of the UK Biobank were genotyped using the Applied Biosystems^™^ UK BiLEVE Axiom^™^ Array or UK Biobank Axiom^™^ Array (14). The genotypes were imputed to the Haplotype Reference Consortium reference panel (24). A total of 25 321 604 SNPs with a minor allele frequency >0.01% and an imputation information score >0.8 were retained. Ethics approval for the UK Biobank study was obtained from the North West Centre for Research Ethics Committee (11/NW/0382). The UK Biobank ethics statement is available at https://www.ukbiobank.ac.uk/the-ethics-and-governance-council/. All UK Biobank participants provided informed consent at recruitment.

**Table 1.** Cohort characteristics

	Female	Male
UK Biobank training set (N = 354 058)		
Sample size (%)	191 361 (54.0)	162 697 (46.0)
Mean height in cm (SD)	162.6 (6.2)	175.8 (6.8)
Mean age (SD)	56.7 (7.9)	57.1 (8.1)
UK Biobank model selection set (N = 6 639)		
Sample size (%)	3635 (54.8)	3004 (45.2)
Mean height in cm (SD)	162.8 (6.2)	175.7 (6.7)
Mean age (SD)	56.5 (7.8)	57.1 (8.1)
UK Biobank test set (N = 81 902)		
Sample size (%)	44 304 (54.1)	37 598 (45.9)
Mean height in cm (SD)	162.6 (6.3)	175.9 (6.8)
Mean age (SD)	56.6 (7.9)	57.1 (8.1)
ALSPAC^*a*^		
Children without mid-parental height^*b*^ (N = 2137)		
Sample size (%)	1343 (62.8)	794 (37.2)
Mean height in cm (SD)	165.9 (6.2)	180.1 (6.7)
Children with mid-parental height (N = 941)		
Sample size (%)	541 (57.5)	400 (42.5)
Mean height in cm (SD)	166.5 (6.0)	180.1 (6.8)

All Individuals had a European ancestry.

^*a*^Only 3078 genotyped children with measured adult height were included.

^*b*^Due to missing data or a parent not being the biological parent.

The ALSPAC cohort initially recruited 14 541 pregnant women who had an expected delivery date between April 1991 and December 1992 in the Bristol and Avon areas in the United Kingdom. Details of this prospective cohort have been described previously (22,23). Children in the ALSPAC cohort were genotyped using the Illumina HumanHap550 quad genotyping platforms and the genotypes were imputed to the 1 000 Genomes Phase 3 reference panel (25). A total of 8932 samples were available for children of European ancestry. Shoeless adult standing height (at age 24 (26)), or individuals who were originally the children in the cohort, as well as shoeless standing height of both biological parents (available for 1532 children) were measured during clinical visits using a Harpenden stadiometer (Holtain Ltd). Among 3078 genotyped children who had measured adult height, 941 had height measured for both biological parents (Table 1), a further 1305 only had measured maternal height, and 151 only had measured paternal height. Shoeless height and weight measures of children during puberty (between ages 8 and 17) was collected from a “Growing and Changing” questionnaire, which was distributed to participants recurrently between September 1999 and February 2010 (27). These questionnaires were completed by either a parent or a child. The children were asked to stand barefoot straight against a wall to mark at the highest point on the head and to measure the distance from the mark on the floor. No specific instruction was provided for measuring weight. Please note that the study website contains details of all the data that are available through a fully searchable data dictionary and variable search tool (http://www.bristol.ac.uk/alspac/researchers/out-data/).

Ethical approval for the study was obtained from the ALSPAC Ethics and Law Committee and the Local Research Ethics Committees. Consent for biological samples has been collected in accordance with the Human Tissue Act (2004). Informed consent for the use of data collected via questionnaires and clinics was obtained from participants following the recommendations of the ALSPAC Ethics and Law Committee at the time.

### Development of a Polygenic Risk Score

**Figure 1.**
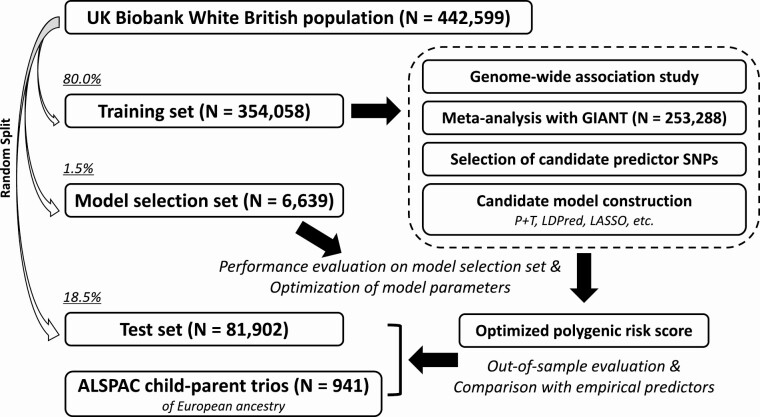
Summary of polygenic risk score development and evaluation of predictive performance.

Details of polygenic risk score construction and initial evaluation are provided in supplementary notes in (28) and summarized in Figure 1. Briefly, we first performed genomewide association study for height in the UK Biobank, based on 354 058 White British ancestry individuals. Next, we meta-analyzed the UK Biobank-based genomewide association study summary statistics with those provided by the GIANT study (21), which included 253 288 European ancestry individuals. We then leveraged a set of state-of-the-art approaches, including linkage disequilibrium-pruning and *P*-value thresholding (29), LDpred (16), and least absolute shrinkage and selection operator (30) regression, to develop a series of new candidate polygenic risk scores based on a random training-model selection-test split in the UK Biobank (Table 1; supplementary notes in (28)). We found that a polygenic risk score generated by least absolute shrinkage and selection operator regression that included 33 938 SNPs demonstrated the best predictive power in the UK Biobank model selection set; thus, we adopted this optimized polygenic risk score for all downstream assessments (supplementary notes in (28)).

### Comparison of Polygenic Risk Score and Mid-Parental Height

We compared the predictive performance of the polygenic risk score and of the mid-parental height in predicting children’s adult height leveraging the ALSPAC cohort. Mid-parental height was calculated as (31)


$$\begin{array}{l} \left(Maternal\;height\;+\;Paternal\;height\right)/2\;-\;6.5\;cm\;for\;girls \end{array}$$

and


$$\begin{array}{ll} & \left(Maternal\;height\;+\;Paternal\;height\right)/2\\ & \;+\;6.5\;cm\;for\;boys. \end{array}$$

We also included comparison with an adapted form of mid-parental height (32):


$$\begin{array}{ll} & 0.75\times \left(Maternal\;height\;+\;Paternal\;height\right)/2\\ & \;+\;37.85\;cm\;for\;girls \end{array}$$

and


$$\begin{array}{ll} & 0.78\times \left(Maternal\;height\;+\;Paternal\;height\right)/2\\ & \;+\;45.99\;cm\;for\;boys. \end{array}$$

This adapted mid-parental height predictor was derived from a Swedish population-based study including 2402 children by fitting sex-specific linear regression models (32).

Polygenic risk scores were calculated for each of the 3078 genotyped children with measured adult height. SNPs that were included in the UK Biobank-based score but were not present in the ALSPAC data were discarded.

We evaluated the predictive performance of these 2 height predictors by proportion of variance explained and the root mean square error (RMSE). We defined those with relative short stature as being among the 2.3% shortest (corresponding to 2 SDs below the mean in the ALSPAC cohort) in females and males, separately. We performed logistic regression and compared predicted to measured height again using the area under the receiver operating characteristic curve (AUROC) and the area under the precision-recall curve (AUPRC). We also tested whether linearly combining the polygenic risk score with mid-parental height or with maternal or paternal height only could significantly improve prediction accuracy by likelihood ratio test of linear regression models and by comparing binary prediction metrics. All assessments included sex-specific analyses.

### Use of Polygenic Risk Score in Khamis-Roche Predictor

In addition, we calculated Khamis-Roche adult height predictors (13), which incorporate a child’s age, current height, current weight, and mid-parental height, for children with self-reported height and weight during puberty. We investigated whether the mid-parental height used in this predictor could be replaced by the genetically predicted height by the polygenic risk score and whether this substitution affected predictive performance.

## Results

### Polygenic Risk Score Achieved Comparable Predictive Performance as Mid-parental Height to Predict Final Adult Height

**Figure 2.**
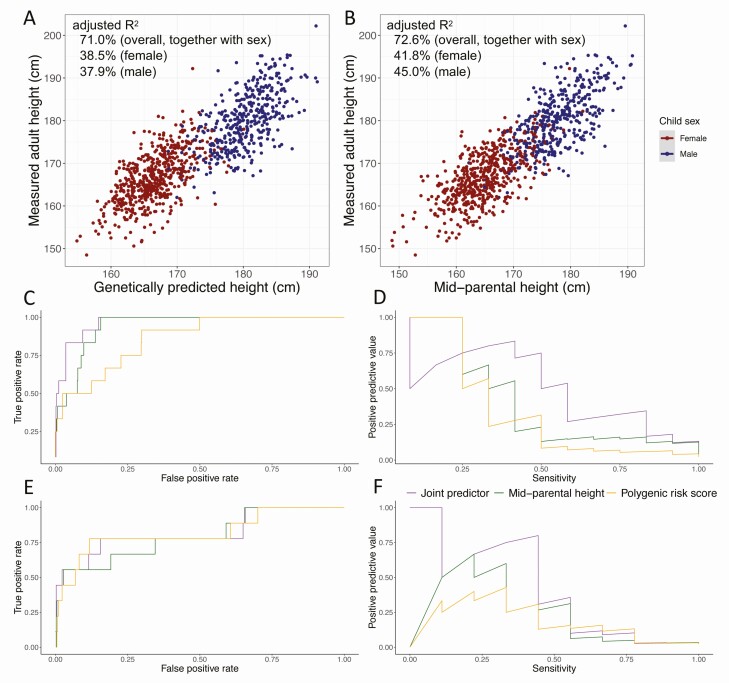
Comparison of predictive performance of the polygenic risk score and the mid-parental height in the ALSPAC cohort (N = 941). (A) The genetically predicted height by the polygenic risk score and (B) the mid-parental height were almost equally correlated with the measured adult height of children. (C) Receiver operating characteristic curve and (D) precision recall curve comparing discriminative power for individuals with short stature of the polygenic risk score, the mid-parental height, and combining these 2 predictors in females. (E) Receiver operating characteristic curve and (F) precision recall curve comparing discriminative power for individuals with short stature of the polygenic risk score, the mid-parental height, and combining these 2 predictors in males.

In the UK Biobank test set consisting of 81 902 individuals (Table 1), in combination with age, sex, recruitment center, genotyping array, and the first 20 genetic principal components (to account for population stratification (14,15,18,33,34)), a polygenic risk score was able to capture 71.1% [95% confidence interval (CI): 70.8%-71.4%] of the total variance in measured adult height (supplementary notes and Supplementary Figure 1 in (28)). In the ALSPAC cohort, among 941 children for whom mid-parental height prediction was available (Table 1), the polygenic risk score, together with sex, explained 71.0% (95% CI: 67.9%-74.1%) of the total variance in adult height (Fig. 2A). This value was similar to the 72.6% (69.6%-75.6%) variance explained by mid-parental height (Fig. 2B) and was consistent in 541 females [adjusted *R*^2^ = 38.5% (95% CI: 32.1%-44.9%) for the polygenic risk score *vs* 41.8% (95% CI: 35.5%-48.1%) for mid-parental height] and 400 males [adjusted R^2^ = 37.9% (95% CI: 30.%5-45.4%) for the polygenic risk score *vs* 45.0% (95% CI: 37.8-52.1%) by mid-parental height]. Meanwhile, the polygenic risk score had an overall prediction RMSE of 4.96 cm, marginally higher than that of the mid-parental height (4.82 cm; Supplementary Figure 2A in (28)).

When predicting adult short stature in a model including sex, the polygenic risk score achieved an AUROC of 0.843 (95% CI: 0.796-0.890) (Supplementary Figure 2B in (28)) and an AUPRC of 0.284 (95% CI: 0.102-0.500) (Supplementary Figure 2C in (28)) to identify children who would have adult short stature. The mid-parental height achieved an AUROC of 0.879 (95% CI: 0.840-0.919) (Supplementary Figure 2B in (28)) and an AUPRC of 0.326 (95% CI: 0.127-0.546) (Supplementary Figure 2C in (28)). Specifically, each SD decrease in the polygenic risk score conferred a 1.62-fold (95% CI: 1.34-2.02) increased odds of having an adulthood short stature for females, with the AUROC being 0.861 (95% CI: 0.814-0.907) (Fig. 2C) and AUPRC being 0.373 (95% CI: 0.087-0.651) (Fig. 2D). The predictive performance was slightly lower for males, as the odds of having an adulthood short stature increased 1.43-fold (95% CI: 1.20-1.75) per SD decrease in the polygenic risk score, with AUROC being 0.820 (95% CI: 0.731-0.909) (Fig. 2E) and AUPRC being 0.181 (95% CI: 0.044-0.518) (Fig. 2F). In contrast, each SD decrease in mid-parental height conferred a 1.53-fold (95% CI: 1.33-1.81) and 1.34-fold (95% CI: 1.15-1.60) increased odds of having an adulthood short stature in females and males, respectively. Its discriminative power was higher than that of the polygenic risk score for females, with higher AUROC (0.943; 95% CI: 0.926-0.960) (Fig. 2C) and AUPRC (0.435; 95% CI: 0.145-0.702) (Fig. 2D). In males, the mid-parental height had a lower AUROC (0.795; 95% CI: 0.709-0.882) (Fig. 2E) and a higher AUPRC than the polygenic risk score (0.237; 95% CI: 0.042-0.653) (Fig. 2F), but both overlapped with those of the polygenic risk score.

Possibly due to population specificity, compared to the ordinary mid-parental height, the adapted mid-parental height predictor (see previous discussion on methods) derived from a Swedish population did not demonstrate superior predictive power [adjusted *R*^2^ = 69.3% (95% CI: 66.1%-72.6%)] (Supplementary Figure 3 in (28)), prediction RMSE = 5.10 cm (Supplementary Figure 2A in (28)), or discriminative power in identifying children at risk for developing adulthood short stature [AUROC = 0.877 (95% CI: 0.837-0.918); AUPRC = 0.291 (95% CI: 0.122-0.539); combined with sex] (Supplementary Figure 2B and 2C in (28)). We therefore did not include this adapted mid-parental height predictor in further analyses.

### A Polygenic Risk Score May Be Combined With Parental Height for Improved Prediction

Often in clinical settings, the height of only 1 parent is known. In the same ALSPAC test set of 941 children, together with sex, a joint predictor combining the polygenic risk score and maternal height achieved an adjusted *R*^2^ of 75.5% (95% CI: 72.7%-78.2%) (Supplementary Figure 4A in (28)) compared to 66.1% (95% CI: 62.6%-69.6%; likelihood ratio test *P*-value = 6.2 × 10^−93^) by the maternal height; a joint predictor combining the polygenic risk score and paternal height achieved an adjusted *R*^2^ of 75.2% (95% CI: 72.5%-78.0%) (Supplementary Figure 4B in (28)) compared to 64.5% (95% CI: 60.9%-68.1%; likelihood ratio test *P*-value = 2.0 × 10^-12^) by the paternal height. When identifying children who would have an adulthood short stature, maternal height, together with sex, had an AUROC of 0.812 (95% CI: 0.776-0.848) and an AUPRC of 0.133 (95% CI: 0.040-0.279); However, combining this with the polygenic risk score improved the AUROC to 0.896 (95% CI: 0.859-0.932) and the AUPRC to 0.322 (95% CI: 0.129-0.524). Similarly, combining the paternal height with the polygenic risk score improved the AUROC from 0.825 (95% CI: 0.772-0.878) to 0.882 (95% CI: 0.834-0.931) and the AUPRC from 0.255 (0.082-0.480) to 0.424 (95% CI: 0.208-0.624). These results were consistent in females and males (Supplementary Figure 4C-4F in (28)).

Furthermore, a joint predictor combining the polygenic risk score and the mid-parental height achieved an adjusted *R*^2^ of 78.5% (95% CI: 76.0%-80.9%), with 55.4% (95% CI: 49.9%-61.0%) in females and 55.9% (95% CI: 49.5%-62.3%) in males (Supplementary Figure 5 in (28)), as well as a substantially lower prediction RMSE of 4.27 cm (Supplementary Figure 2A in (28)). This improvement was significant over using the polygenic risk score (likelihood ratio test *P*-value = 6.3 × 10^−73^) or the mid-parental height (likelihood ratio test *P*-value = 5.9 × 10^−58^) alone. The ability to identify individuals with an adulthood short stature was also improved with an AUROC of 0.904 (95% CI: 0.862-0.947) (Supplementary Figure 2B in (28)) and an AUPRC of 0.490 (95% CI: 0.254-0.710) (Supplementary Figure 2C in (28)), in models including sex. Specifically, the AUROC increased to 0.969 (95% CI: 0.955-0.983) in females (Fig. 2C) and 0.821 (95% CI: 731-0.911) in males (Fig. 2E); the AUPRC increased to 0.530 (95% CI: 0.227-0.824) in females (Fig. 2D) and 0.407 (95% CI: 0.067-0.772) in males (Fig. 2F).

### Polygenic Risk Score Could Substitute Mid-Parental Height in Khamis-Roche Approach for Improved Prediction

**Figure 3.**
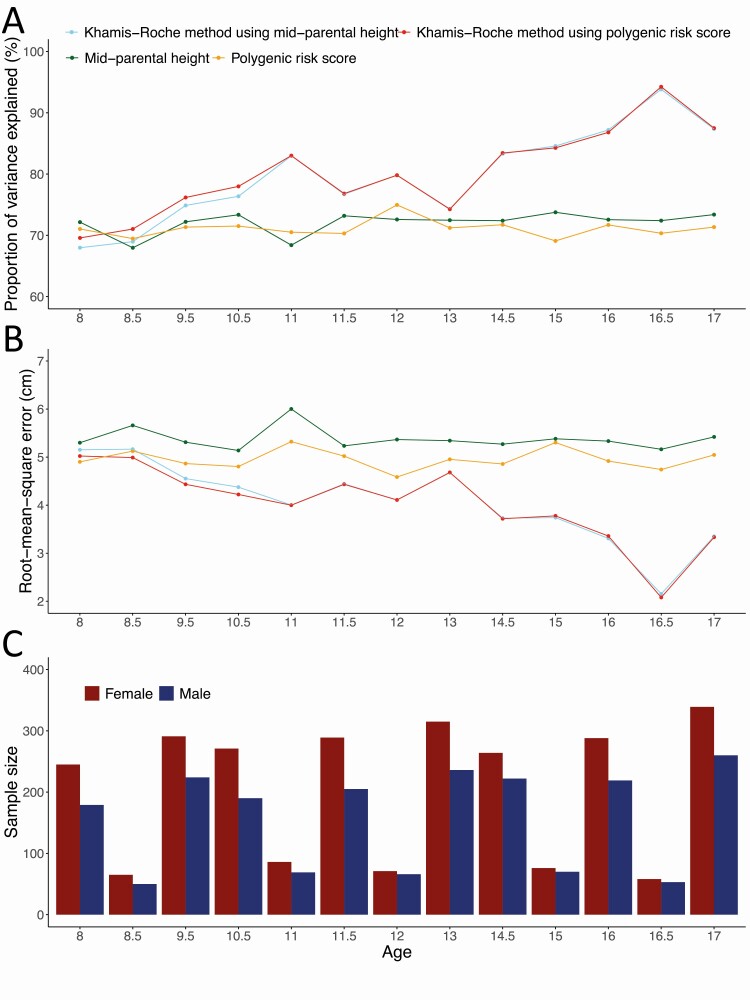
The polygenic risk score may quantify genetic contribution in Khamis-Roche method in place of the mid-parental height. (A) Proportion of variance explained and (B) prediction root mean square error by Khamis-Roche predictors as well as the polygenic risk score and the mid-parental height are compared based on children with available pubertal development information in the ALSPAC cohort. (C) Sample sizes at ages 8 to 17. Time points at which both females and males had ≥50 samples were retained. Children’s chronological age at each survey was rounded to the nearest 0.5 to generate Khamis-Roche predictors. Sex-specific analyses are summarized in Supplementary Figure 6 in (28).

Khamis-Roche adult height predictors were derivable for varying sample sizes at 13 time points (Fig. 3). At ages 8, 8.5, 9.5, and 10.5, we tested the performance of substituting mid-parental height with the polygenic risk score in the Khamis-Roche approach. We found that this led to marginally higher proportion of variance explained in adult height as well as slightly lower prediction RMSE (Fig. 3A and 3B). At ages ≥11 years, the 2 Khamis-Roche predictors displayed no distinguishable difference. These findings were consistent in females and males, respectively (Supplementary Figure 6 in (28)). Although underpowered by the limited number of short stature cases, the polygenic risk score-based Khamis-Roche predictor and the mid-parental height-based Khamis-Roche predictor had highly consistent discriminative power in identifying children who would have a short stature in adulthood (Supplementary Figure 7 in (28)).

## Discussion

Accurate prediction of adult height is important for the management of short stature in childhood. As well as selecting which children may require growth hormone therapy for idiopathic short stature (1-3), it may also help to inform which children should be further investigated for chronic health conditions affecting growth, such as growth hormone deficiency or genetic syndromes (35). Given the exceptionally high heritability of height, there have been long-standing interest and efforts to genetically predict adult height. Yet, the most commonly used genetic predictor has been the mid-parental height, which simply averages the height of a child’s biological parents and adjusts for the sex of the child. In this study, we constructed efficient polygenic risk scores for adult height, leveraging > 600 000 individuals of European ancestry. Using an external cohort of 941 samples, we demonstrated that, for the first time, a polygenic risk score was able to perform as well as the mid-parental height in predicting children’s adult height and in identifying children who would have adult short stature. Further, combining mid-parental height and the polygenic risk score provided better prediction accuracy than either metric alone.

The polygenic risk score might provide clinical utility. For example, parental height may not be available due to various reasons (eg, many children do not know both parents’ height), and in many more cases it is inaccurately measured or reported, and thus a potentially erroneous estimate might be supplied. Also, large discrepancies in parental heights render mid-parental height a less valid adult height predictor, particularly when some parents do not achieve their genetically determined height because of disease that is not inherited by the child. Under these circumstances, a polygenic risk score may be utilized instead, not only as a single predictor but also in other accurate multivariate predictive models, such as the Khamis-Roche method (13). Even though the self-reported height and weight measures during puberty in the ALSPAC cohort may contain measurement errors, the superior performance of Khamis-Roche predictors over the mid-parental height or the polygenic risk score alone should encourage further investigations into incorporating an accurate genetic predictor into established scoring schemes. We further demonstrated that even if only 1 parent could provide measured height, a combined predictor with the polygenic risk score could have importantly improved predictive and discriminative power over using the single parental height. We posit that these advantages could be considered when undertaking genetic prediction of adult height both in both clinical and research settings. Since genomewide genotyping has become more affordable (currently priced at approximately US$40 in a research context), is undertaken once in a lifetime, and could be used to predict several health outcomes, genetic prediction of adult height in children and adolescents could be widely and cost-effectively applied in research and clinical practice.

Combining the polygenic risk score and the mid-parental height could enhance predictive performance. Intuitively, the mid-parental height, while being a crude estimate of the genetic contribution, may additionally capture variance due to unmeasured environmental exposures shared by the parents and the children, particularly when families in a target population live in the same geographical area. This may partially explain the decrease of accuracy in mid-parental height prediction when a large discrepancy exists in the parental height of a child. On the contrary, our polygenic risk score more accurately quantifies the genetic contribution toward height without measuring environmental exposures. Hence, the 2 predictors provide non-overlapping information. However, how proper calibration should be carried out and whether a joint predictor is generalizable will require extensive exploration in future studies.

Our study has important limitations. First, our findings should be considered population-specific. Polygenic risk scores are usually not directly transferrable to a population of a different ethnic background (36-39). Population-specific polygenic risk scores should be developed to further explore their utility for height prediction, especially among non-European populations—once again underlining the importance of larger genetic studies of non-European ancestry that are currently scant. It should also be noted that the adult height of children in ALSPAC was, on average, 3 to 4 cm taller than that in the UK Biobank, because the former represents a much younger population. We therefore adopted the definition of short stature as 2 SDs below population mean, instead of referring to a general population-based growth curve. Moreover, despite a high heritability, adult height is also influenced by a wide range of genetic and nongenetic factors not included in the polygenic risk score, including but not limited to chronic conditions retarding growth (eg, genetic syndromes, such as Turner (40-42) and Prader-Willi syndromes (43-45), treatment for childhood cancer (46, 47), acquired growth hormone deficiency secondary to head trauma (35), etc.), rare pathogenic variants of large effects in pivotal genes (48-50), and long-term medication (eg, use of inhaled corticosteroids (51-53)). Whether any interplay exists between the polygenic risk score and these risk factors may be tested with larger sample sizes and by testing the performance of this polygenic risk score in pediatric populations at risk for compromised growth.

Lastly, although not measured in our study cohorts, predictions based on current height and bone age remain powerful indicators of final adult height. In fact, in a few cases where bone age-based predictors were evaluated, the proportion of variance explained by a combination of current height, chronological age, and bone age exceeded 60% at age 3 in both boys and girls (54) and continued increasing toward >99% by age 17, while the mid-parental height or the polygenic risk score alone could only account for approximately 40% of the total variance in sex-specific analyses. Nevertheless, it has been suggested that bone age-based predictors (eg, the Tanner-Whitehouse predictors) might also benefit from adding a genetic predictor, particularly for children with an extremely short or tall stature (54). Notably, a genetically predicted height is not sensitive to pubertal patterns (early or late puberty), which typically affect bone maturation, and this predictor could be computed as early as at birth, whereas adult height predictions based on bone age are not precise before the age of 3 years. For all the previously discussed reasons and given the decreasing costs and increased accessibility of genomewide genotyping, we anticipate that a genetically predicted height may play a larger role in informing clinical decisions in the future.

## Conclusions

In summary, we generated a polygenic risk score and demonstrated that it could successfully predict adult height in a population of healthy children. Our findings support the use of this genetic predictor in certain settings as an alternative or in combination with traditional adult height predictors, such as mid-parental height, and possibly also with bone age-based methods to enhance screening for children at risk for short stature in adulthood.

## Data Availability

Some or all data generated or analyzed during this study are included in this published article or in the data repositories listed in references. TL had full access to all the data in the study and take responsibility for the integrity of the data and the accuracy of the data analysis. Data from the UK Biobank and the ALSPAC are available upon successful project application to the respective research committees. Scoring files (including variants, effect alleles, and weights) of the polygenic risk scores have been deposited on the PGS catalog (https://www.pgscatalog.org/score/PGS000758/). Restrictions apply to the availability of some or all data generated or analyzed during this study to preserve patient confidentiality or because they were used under license. The corresponding author will on request detail the restrictions and any conditions under which access to some data may be provided.

## Additional Information

***Disclosure Summary:*** JBR has served as a consultant to GlaxoSmithKline and Deerfield Capital for their genetics programs. The other authors have no relevant disclosures.
